# Rare, potentially pathogenic variants in 21 keratoconus candidate genes are not enriched in cases in a large Australian cohort of European descent

**DOI:** 10.1371/journal.pone.0199178

**Published:** 2018-06-20

**Authors:** Sionne E. M. Lucas, Tiger Zhou, Nicholas B. Blackburn, Richard A. Mills, Jonathan Ellis, Paul Leo, Emmanuelle Souzeau, Bronwyn Ridge, Jac C. Charlesworth, Richard Lindsay, Jamie E. Craig, Kathryn P. Burdon

**Affiliations:** 1 Menzies Institute for Medical Research, University of Tasmania, Hobart, Tasmania, Australia; 2 Department of Ophthalmology, Flinders University, Adelaide, South Australia, Australia; 3 South Texas Diabetes and Obesity Institute, Department of Human Genetics, School of Medicine, University of Texas Rio Grande Valley, Brownsville, Texas, United States of America; 4 Queensland University of Technology and Translational Research Institute, Princess Alexandra Hospital, Brisbane, Queensland, Australia; 5 Institute of Health and Biomedical Innovation, Queensland University of Technology, Brisbane, Queensland, Australia; 6 Richard Lindsay and Associates, East Melbourne, Victoria, Australia; University of Florida, UNITED STATES

## Abstract

Many genes have been suggested as candidate genes for keratoconus based on their function, their proximity to associated polymorphisms or due to the identification of putative causative variants within the gene. However, very few of these genes have been assessed for rare variation in keratoconus more broadly. In contrast, *VSX1* and *SOD1* have been widely assessed, however, the vast majority of studies have been small and the findings conflicting. In a cohort of Australians of European descent, consisting of 385 keratoconus cases and 396 controls, we screened 21 keratoconus candidate genes: *BANP*, *CAST*, *COL4A3*, *COL4A4*, *COL5A1*, *FOXO1*, *FNDC3B*, *HGF*, *IL1A*, *IL1B*, *ILRN*, *IMMP2L*, *MPDZ*, *NFIB*, *RAB3GAP1*, *RAD51*, *RXRA*, *SLC4A11*, *SOD1*, *TF* and *VSX1*. The candidate genes were sequenced in these individuals by either whole exome sequencing or targeted gene sequencing. Variants were filtered to identify rare (minor allele frequency <1%), potentially pathogenic variants. A total of 164 such variants were identified across the two groups with no variants fulfilling these criteria in cases in *IL1RN*, *BANP*, *IL1B*, *RAD51* or *SOD1*. The frequency of variants was compared between cases and controls using chi-square or Fishers’ Exact tests for each gene with at least one rare potentially pathogenic variant identified in the case cohort. The number of rare potentially pathogenic variants per gene ranged from three (*RXRA*) to 102 (*MPDZ*), however for all genes, there was no difference in the frequency between the cases and controls. We conclude that rare potentially pathogenic variation in the 21 candidate genes assessed do not play a major role in keratoconus susceptibility and pathogenesis.

## Introduction

Keratoconus (OMIM 148300) is a complex disease characterised by progressive stromal thinning and conical protrusion of the cornea which usually develops in the second decade of life. These abnormalities can lead to high myopia and irregular astigmatism which cause severe visual impairment and affect patients during their most productive years with quality of life estimates similar to that of age related macular degeneration.[1] The global incidence of keratoconus is approximately 1 in 50,000,[2, 3] and the prevalence varies greatly between ethnicities and geographical locations, with a prevalence in Caucasians of between 54.5 and 265 per 100,000 individuals.[2–4] While there is strong evidence of the role of genetics in keratoconus susceptibility, only a few specific genetic risk factors have been identified. Key challenges include the complex nature of keratoconus, the genetic heterogeneity and adequate sample sizes for well powered genetic analyses. Identifying genes and biological pathways involved in keratoconus pathogenesis is critical for the development of novel treatments and biomarkers to aid early diagnosis, which together would substantially improve the quality of life of individuals with keratoconus. Several approaches have been used to elucidate genetic variants that underpin keratoconus susceptibility.

Linkage analysis in extended families has identified more than 20 linkage regions.[5–21] However, only regions on chromosome 5q have been replicated.[5, 8, 16, 19, 20] The number of loci identified highlights the heterogeneous nature of the disease. Such family-based studies have implicated few candidate variants and genes to date, due to the size of the regions. The most promising keratoconus gene identified with this method is *mir184*. This microRNA gene was found to have a pathogenic variant within the DNA binding domain in a family from Northern Ireland.[13, 14, 22] The same variant was subsequently identified in an unrelated family from Spain[8] and similar variants predicted to reduce the stability of the miRNA secondary structures were identified in two sporadic cases.[23] It is however important to note that these individuals had both keratoconus and congenital cataract and therefore have a more heterogeneous phenotype. Variants in other genes, such as *IL1RN* and *SLC4A11*, have been hypothesised to play a role in disease due to the linkage-based identification of potentially pathogenic variants in an Ecuadorian family.[18] *IL1RN* which encodes IL1 receptor antagonist and *SLC4A11* which encodes sodium bicarbonate transporter-like protein 11 were selected as candidate genes due to their involvement in the immune response and apoptosis, respectively. However, these genes have not been assessed in other cohorts of keratoconus patients.

Three genome-wide association studies (GWAS) have identified four genome-wide significant loci as well as several loci that show a suggestive association with keratoconus. A large GWAS which assessed loci associated with central corneal thickness in keratoconus patients identified two single nucleotide polymorphisms (SNPs) associated with keratoconus in intronic regions of *FOXO1* (rs2721051) and *FND3CB* (rs4894535).[24] The same study found a suggestive association at rs1324183, between *MPDZ* and *NFIB*, which reached genome-wide significance following replication and meta-analysis.[24, 25] Similarly, rs4954218, upstream of *RAB3GAP1*, showed a suggestive association in the initial study, but reached significance after replication and meta-analysis.[26, 27] Other suggestive associations include SNPs in the promoter of *HGF*,[28, 29] rs1536482 between *RXRA* and *COL5A1*,[24] rs9938149 between *BANP* and *ZNF469*,[24] and two intronic SNPs (rs757219 and rs214884) in *IMMP2L*.[27] The identification of these loci has provided important insights into keratoconus genetics, however, functional variation at these loci have not yet been determined. While the most significant SNPs are located in non-coding regions, many of the nearby genes make good biological candidates for keratoconus. Thus, we hypothesise that rare coding variation in these genes may be involved in keratoconus susceptibility.

Many genes have also been hypothesised to play a role in keratoconus based on their function and known corneal expression. The genes selected in the present study can broadly be categorised as regulatory genes, such as *CAST*[30] and *VSX1*;[31] structural genes, including the collagen genes *COL4A3* and *COL4A4*;[32] and genes involved in immune responses, such as *SOD1*,[33] *TF*[34] and *RAD51*,[35] *IL1A*[36] and *IL1B*.[37] The initial studies that implicated *CAST*, *COL4A3*, *COL4A1*, *TF*, *RAD51*, *IL1A* and *IL1B* in keratoconus showed associations at nearby or intronic SNPs. Therefore, the supporting evidence of the involvement of these genes in disease has both a biological-, and positional-, basis. In contrast, the genes *VSX1* and *SOD1*, were initially proposed to play a role in keratoconus due to the identification of sequence variants in keratoconus patients that were absent in controls. As the first gene postulated to contribute to keratoconus, *VSX1* has been extensively assessed in many populations with conflicting results. Many studies conclude that *VSX1* is likely to be involved in keratoconus pathogenesis,[31, 36, 38–49] while a similar number of studies do not find evidence of association.[50–63] Similarly, the superoxide dismutase gene (*SOD1*) has been screened in several populations including Slovenian,[53] Iranian,[45, 63] Italian,[43] Greek,[59] Saudi Arabian[64] and multiethnic[33] cohorts. A 7bp intronic deletion was observed in cases but not controls in two of these reports[33, 43] and was significantly more frequent in cases compared to controls in another,[59] however, the remaining studies did not observe the variant.[45, 53, 63, 64] Given the contention surrounding the involvement of *VSX1* and *SOD1*, and the few studies that assessed the remaining functional candidates, further analysis is required to determine if they contribute to keratoconus susceptibility and pathogenicity.

Through these different methodologies and approaches, many candidate genes have been hypothesised to play a role in keratoconus based on their function, their proximity to associated SNPs or due to the identification of putative causative variants within the gene. However, the majority of these genes have not been assessed beyond the initial study, and for those that have, the majority of studies have been small, with fewer than 100 keratoconus cases. To address this, our study assessed the role of 21 candidate genes in the largest cohort of keratoconus cases to date (n = 385), compared to 396 population controls. Specifically, our study examines the frequency of potentially pathogenic variants in *MPDZ*, *RXRA*, *RAB3GAP1*, *FOXO1*, *BANP*, *HGF*, *COL5A1*, *IMMP2L*, *FNDC3B*, *NFIB*, *ILRN*, *SLC4A11*, *CAST*, *COL4A3*, *COL4A4*, *TF*, *SOD1*, *VSX1*, *RAD51*, *IL1A* and *IL1B* in a cohort of Australians of European descent. We have previously assessed potentially pathogenic variants in *ZNF469* in our cohort.[65]

## Materials and methods

### Study participants

All investigations adhered to the principles of the Declaration of Helsinki and were approved by the Southern Adelaide Clinical Human Research Committee and the Human Research Ethics Committee Tasmania, with all participants giving written informed consent.

The case cohort consisted of 385 keratoconus patients of European descent. These individuals were recruited through the Flinders Eye Clinic (Adelaide, Australia), following referral by their treating optometrist and ophthalmologists or were recruited from across Australia via mail through Keratoconus Australia. All clinical examinations were performed by an experienced ophthalmologist. Individuals were diagnosed with keratoconus if they had a history of corneal transplantation for keratoconus, had videokeratographic features of keratoconus, or any of the following signs: conical corneal protrusion, central or paracentral stromal thinning or other distinctive features such as Fleischer’s ring, Vogt’s striae, epithelial or sub-epithelial scarring, or oil droplet sign and/or scissoring of the retinoscopic reflex.

The control cohort consisted of 396 ethnically matched females from the Anglo-Australasian Osteoporosis Genetics Consortium. These individuals are known to have moderately high, or low, bone mineral density measurements (1.5<|BMD|<4.0), however were not examined for eye disease. This cohort has been previously described in detail.[66]

### Sequencing data

Whole exome sequencing (WES) data were available for 99 keratoconus cases. WES was conducted by Macrogen Inc. using the SureSelect Human All Exon V4 enrichment kits (Agilent Technologies Inc., Santa Clara, CA, United States of America) with paired-end sequencing on an Illumina HiSeq 2000 (Illumina, San Diego, CA, United States of America). The Churchill pipeline[67] was used to align raw reads to hg19 using BWA-MEM (version 0.7.12)[68] and variants were joint-called with SAMtools and BCFtools (versions 1.3.1).[69]

In addition, the 21 genes of interest were assessed using a targeted sequencing approach in 341 cases using the HaloPlex Target Enrichment System (Agilent) with a custom designed probe panel using pooled DNA samples. This method is described in detail previously.[65] In brief, DNA pools containing equimolar DNA samples from eight keratoconus patients were prepared and indexed with a unique primer cassette during the library preparation. Sequencing was conducted in batches of 11 pools using MiSeq V2 Reagent kits (300 cycles) with paired-end reads on an Illumina MiSeq. Agilent’s SureCall program was used to align raw reads to hg19 using BWA-MEM and Agilent’s SNPPET SNP Caller was used to call variants. Due to the DNA pooling, it was expected that if one alternate allele was present in a single pool, it would be observed in approximately 6.25% of reads. Therefore, the minimum allele frequency for heterozygous single nucleotide variants (SNV) was set to 0.035. To allow for comparison of the sequencing methods, DNA from 55 cases were including in both the WES and targeted sequencing dataset, however variants identified in these samples were only counted in analyses once.

For the control cohort (n = 396), WES was generated using Illumina’s TruSeq Exome Enrichment on an Illumina HiSeq 2000 at the University of Queensland Centre for Clinical Genomics. Raw reads were aligned to hg19 using novoalign (version 02.08) and variant calling and quality score calibration was conducted using GATK[70] (version 3.2–2), according to GATK’s ‘Best Practices Guidelines’.[71, 72]

### Included genomic regions and variant annotation

To ensure a sound comparison when comparing the frequency of variants between data with different capture methods, only target regions that were common to all three capture methods (both exome captures and the targeted sequencing) with a mean read depth ≥10 were included in the analysis. For the WES data, all included individuals had high confidence genotypes for ≥90% of the included regions. A complete list of the included regions is available in S1 Table. Coverage of genes are reported for the coding regions of the longest transcript included in the captures (S2 Table). However, other regions, including noncoding regions, may have also been included in analysis.

Using ANNOVAR,[73] the variants identified within the included regions were annotated with the minor allele frequency (MAF) observed in the non-Finnish European population of the Exome Aggregation Consortium database (ExAC NFE),[74] pathogenicity/deleteriousness predictions from Sorting Tolerant from Intolerant (SIFT)[75], the HumDIV algorithm from Polymorphism Phenotyping v2 (PolyPhen2)[76] and Combined Annotation–Dependent Depletion v1.3 (CADD).[77]

### Filtering strategy to identify potentially pathogenic variants

Variants were only included in analyses if a sequencing depth of ≥10 and a quality score of ≥20 was obtained. Variants meeting these thresholds were then filtered to include SNVs that were predicted to be pathogenic by SIFT or PolyPhen2 with a MAF <0.01. In addition, SNVs with a CADD score ≥15 that met the MAF threshold were included in this filtering strategy. These variants were considered ‘potentially pathogenic variants’ and were included in our statistical analysis. Insertions and deletions were not included in the analysis.

### Variant validation by direct sequencing

To validate variants identified in the case cohort, primers were designed using PrimerBlast.[78] DNA was amplified with MyTaq HS Mix (Bioline, London, UK) and purified using either Agencourt AMPure XP (Beckman Coulter, Brea, CA, United States) according to the manufacturer’s instructions, or equivalent magnetic beads prepared in-house. Purified amplicons were sequenced using the BigDye Terminator v3.1 Cycle Sequencing Kit (Applied Biosystems, Foster City, CA, United States) on an ABI 3500 (Applied Biosystems). DNA sequences were aligned to the human reference genome (hg19), and chromatograms were manually inspected at the position of each variant using Sequencher 4.10.1 (Gene Codes Corporation, Ann Arbor, MI, USA) to assess validation.

### Statistical analysis

Following variant filtering, genes with at least one alternate allele identified in the case cohort were included in the statistical analysis. For each of these genes separately, the number of variants that fulfilled the filtering strategy was compared between cases and controls using a Yates corrected chi squared test or a Fishers’ exact test, where appropriate. Odds ratios and 95% confidence intervals were also calculated. As 21 genes were assessed in the present study, a significance threshold of p <0.0024 (0.05/21) was determined using the Bonferroni correction for multiple testing.

## Results

Demographic details for keratoconus cases and controls are provided in Table 1.

**Table 1:** Demographics of keratoconus cases and controls at the time of examination, where n = the number of individuals and age is reported in years.

Cohort	n	Mean age (range)	% Female	Disease status
cases	385	45.2 (14–85)	44.2	affected
controls	396	69.7 (46–86)	100	unscreened

For each gene the coverage statistics are summarised for the coding portions of the longest transcript that best fit the capture designs (S2 Table). Due to a high GC content, the coverage of *BANP* was poor, particularly in the control data and the targeted sequencing dataset. When considering regions captured by probes across the three datasets, only 58% of the gene was captured, however, 70% of these regions had sufficient coverage for analysis. Similarly, 77.4% of *VSX1* was captured across all datasets due to insufficient capture of GC-rich regions and 60.6% of these captured regions met the coverage threshold for inclusion in the analysis. Despite this poor coverage, a number of previously reported *VSX1* variants in keratoconus were sufficiently covered for analysis. The captured regions of the remaining genes ranged from 75.3–97.5% and, apart from *FOXO1*, 99% - 100% of the coding bases in captured regions were included in analysis (S2 Table). For *FOXO1*, the included portion of the captured region dropped to 82.5% as part of the first exon did not meet the minimum depth threshold for variant calling in the WES datasets. It is important to note that additional regions, including non-coding regions, may also have been included in analysis.

For the 55 individuals that were included in both the WES and targeted sequencing datasets, a total of 20 potentially pathogenic variants were observed 21 times in the WES data. Of these variants, 18 were also called in the targeted sequencing data, though were only included once in analysis. The remaining three variants were not called in the targeted sequencing data as they did not meet the minimum inclusion threshold of 3.5% of reads. However, the alternate allele threshold of 3.5% was carefully selected to minimise the inclusion of spurious variant calls.

Following variant filtering, 164 potentially pathogenic variants were identified across both cases and controls (S3 Table). This included 138 nonsynonymous variants, 21 synonymous variants, three nonsense variants, one intronic variant and one variant in a 3’ untranslated region. Of these variants, 70 were unique to the cases, 69 were only observed in controls and 25 were identified in both groups. For the cases, a total of 146 potentially pathogenic variants were observed, while 192 were identified in controls. Two variants, p.(R85Q) in *IL1A* and p.(P1379S) in *COL5A1*, were each observed in the homozygous state in a single control. All other variants were observed as heterozygotes.

No potentially pathogenic variants were identified in *IL1RN* in either cases or controls. Additionally, potentially pathogenic variants were not observed in the case cohort in *BANP*, *IL1B*, *RAD51* or *SOD1*. However, in the control group, one potentially pathogenic variant was observed in both *IL1B* and *SOD1*, two variants were identified in *BANP*, and three were observed in *RAD51*. For the remaining genes, the total number of potentially pathogenic variants identified across both groups ranged from three in *RXRA* and up to 102 in *MPDZ* (Table 2). Of the genes included in the chi-square or Fishers’ exact tests, *COL4A3* and *MPDZ* showed a nominally higher frequency of potentially pathogenic variants in controls compared to the case cohort, with both genes obtaining a p <0.05 in the burden analysis, however, neither gene remained significant under correction for multiple testing. All other genes showed no difference between groups.

**Table 2:** Burden test results for genes in which at least one potentially pathogenic variant was identified in the case cohort. For each gene, the number of alternate (alt.) alleles (potentially pathogenic variants) and wild type alleles (WT) are shown for both cases and controls, along with the p-value (P), odds ratio (OR) and the 95% confidence interval (CI).

	Case Alleles		Control Alleles				
Gene	Alt.	WT	Alt.	WT	P	OR	95% CI
*CAST*	6	764	7	785	>0.999*	0.881	0.262–2.924
*COL4A3*	21	749	39	753	0.024*	0.541	0.305–0.957
*COL4A4*	7	763	9	783	0.846*	0.798	0.267–2.348
*COL5A1*	32	738	22	770	0.176*	1.518	0.846–2.731
*FNDC3B*	5	765	2	790	0.281	2.582	0.446–19.237
*FOXO1*	3	767	1	791	0.368	3.094	0.289–77.330
*HGF*	3	767	6	786	0.507	0.512	0.101–2.302
*IL1A*	3	767	8	784	0.225	0.383	0.080–1.587
*IMMP2L*	1	769	3	789	0.625	0.342	0.014–3.665
*MPDZ*	40	730	62	730	0.045*	0.645	0.419–0.991
*NFIB*	3	767	3	789	>0.999	1.029	0.166–6.375
*RAB3GAP1*	5	765	5	787	>0.999	1.029	0.258–4.101
*RXRA*	1	769	2	790	>0.999	0.514	0.018–7.195
*SLC4A11*	4	766	6	786	0.753	0.684	0.162–2.739
*TF*	8	762	9	783	>0.999*	0.913	0.320–2.591
*VSX1*	4	766	1	791	0.212	4.131	0.438–97.260

P-values obtained using a Yates corrected chi-square test are denoted by a *. All other p-values were obtained using a Fisher’s exact test. P-values <0.0024 were considered significant.

## Discussion

We show that rare potentially pathogenic variants in 21 candidate genes were not enriched in keratoconus in our large cohort of Australians of European descent. We identified a total of 164 potentially pathogenic variants across cases and controls in 20 of the 21 genes assessed, however, variants fulfilling these criteria were equally common between the two groups. No potentially pathogenic variants were identified in *IL1RN* in either cases or controls, suggesting that this gene is highly conserved. Additionally, no potentially pathogenic variants were observed in our cases in *BANP*, *IL1B*, *RAD51* or *SOD1*. Based on these findings, we suggest that rare potentially pathogenic variants in the 21 genes assessed do not contribute to keratoconus development in our cohort.

We assessed candidate genes in a large cohort of Australian keratoconus cases by combining WES data and targeted gene sequencing using pooled DNA samples. We have previously demonstrated the utility of this approach.[65] To ensure high quality variant calls, particularly those identified in targeted sequencing method, the present study only included SNVs. Extensive variant validation experiments were conducted to confirm real variants and exclude sequencing artefacts. Furthermore, the variant calls for the individuals included in both the WES and targeted sequencing datasets were compared to assess concordance. In these individuals, three variants identified in the WES data did not reach the inclusion thresholds in the targeted sequencing data in the present study, which is consistent with previous findings. Additionally, in our previous study we observed a variant in the targeted sequencing data that did not meet the inclusion thresholds for the WES data.[65] Overall we demonstrated a high concordance of rare variant calls between the two datasets.

For the few genes that have previously been assessed, *in silico* tools that predict the pathogenicity of variants such as PolyPhen2 and SIFT have been used to help differentiate between benign variants and potentially pathogenic variants. However, these algorithms can only assess nonsynonymous variants. To allow for the inclusion of potentially pathogenic synonymous and non-coding variants in our study, variants were also scored with CADD. CADD uses a machine learning method to predict the deleteriousness of variants such that a scaled score above 10 refers to the top 10% of variants ranked by deleteriousness, a scaled score of 20 or above includes the top 1% of variants and so on.[77] In the present study, a scaled CADD score of 15 was selected as the minimum threshold for synonymous and non-coding variants. In contrast, nonsynonymous variants were included if they were predicted to be damaging by SIFT, or damaging/possibly or probably damaging by PolyPhen2 or obtained a CADD scaled score of ≥15. This broad definition of potentially pathogenic variants was designed to minimise the exclusion of likely important disease-related variants.

We compared the frequency of potentially pathogenic variants between Australian keratoconus cases and controls, using a control cohort consisting of 396 females. These individuals were not screened for keratoconus, however, considering the prevalence of keratoconus in Caucasians is between approximately 1 in 375 and 1 in 2000[2–4] it is unlikely that more than one or two individuals in this cohort have keratoconus, if any. Considering the large sample size for both the case and control cohorts, this is unlikely to affect our findings. Additionally, the controls were 100% female while 44.2% of the cases were female. This is a potential limitation, however, while epidemiological studies report a slightly higher prevalence in males compared to females in Caucasian populations, these differences are not significant.[2–4] Moreover, all of the genes assessed in the present study are autosomal. Consequently, we do not expect sex-based differences in the frequency of variants, and therefore, the use of this all-female cohort is unlikely to affect the outcomes of the present study.

Gaps in coverage, occurring either at the probe design stage or at the sequencing phase, were a limitation of this study. Similarly, the capture methods were specifically designed to capture exonic regions, and although non-coding variants were not excluded, very few were observed. In addition, insertion and deletion variants were not assessed due to the challenges of calling such variants in the targeted sequencing using pooled DNA. These limitations mean that some variants will have been missed, but this is not expected to be a major bias between cases and controls due to limiting the analysis to regions adequately covered by all methods. Some specific variants that have previously been reported in keratoconus could not be assessed. This is particularly important for *SOD1*, as the variant previously associated with keratoconus was a 7bp intronic deletion, outside of the capture regions in this study. Similarly, *IL1RN* and *SLC4A11* were implicated by an intronic SNV and a 54 bp intronic deletion (respectively) which almost completely co-segregated with keratoconus in an Ecuadorian family.[18] Although these specific variants could not be assessed in our study, the overarching design and aim was to examine the coding regions of the 21 selected genes for enrichment of potentially pathogenic variants in keratoconus.

*VSX1*, which encodes a vertebrate paired-like homeodomain transcription factor with known ocular expression,[79, 80] is the most studied gene in keratoconus. It was initially studied as a candidate gene for posterior polymorphous dystrophy (PPD; OMIM 122000) as it was located within a linkage region for this disease.[31, 81] PPD is a rare, bilateral corneal dystrophy that primarily affects the endothelium and results in variable degrees of visual impairment which has been associated with keratoconus.[82–86] It was therefore hypothesised that the two diseases may share a common genetic basis. The original paper identified four *VSX1* variants in keratoconus cases that were absent in 277 controls,[31] as well as p.(G160D) and p.(P247R) in a family with PPD.[31] Subsequently, the p.(G160D) variant has been identified in keratoconus cases in two Italian studies[38, 43] a European cohort,[42] and in two cases in the current study. This was the only variant observed in our cases that had been reported in other keratoconus cases. In contrast, the p.(G160D) variant was found at similar frequencies in both cases and controls in a Han Chinese cohort,[36] indicating that this variant is not highly penetrant for keratoconus, at least in the Chinese population. Interestingly, the p.(G160V) variant, which results in a different amino acid substitution at the same position, has been observed in cases in two Korean studies.[40, 57] The p.(P247R) variant originally reported in the PPD family[31] and subsequently reported in keratoconus[38, 43, 51] was observed in a single control subject in our study. As our controls were not screened for eye disease it is possible that a small number of individuals in this group may have keratoconus or PPD and therefore the involvement of this variant in disease cannot be ruled out. Furthermore, as keratoconus is a complex disease it is likely that unaffected individuals may carry risk alleles, without ever developing disease, therefore, to assess the potential role of specific variants such as p.(P247R), a large meta-analysis is required. Additionally, we identified p.(L237P) and p.(E234K) for the first time in keratoconus, each observed in a single case. Taken together, the VSX1 gene may contribute in a very small number of cases with clear segregation in families identified,[38, 39, 45, 47] however, rare potentially pathogenic variants in this gene do not contribute widely to keratoconus susceptibility in our cohort.

## Conclusion

In conclusion, our study demonstrated that overall the frequency of potentially pathogenic single nucleotide variants is not different between cases and controls in 21 candidate genes in our large cohort of Australians of European descent. While specific variants may contribute to keratoconus risk in a small proportion of cases, these genes do not contribute to disease in the vast majority of keratoconus patients. With the frequency of the variants observed in the present study, much larger cohorts are required for individual variant analysis with sufficient power. As demonstrated by the success of identifying the keratoconus-associated gene *mir184*,[13, 14, 22] family studies paired with next-generation sequencing technologies may be a powerful method for elucidating specific disease-causing variants. Together, this would allow for less biased approaches for variant identification without a priori hypothesis and candidate gene selection. Furthermore, our study suggested that coding variants in the closest genes to GWAS hits are unlikely to contribute to disease. Therefore, fine-mapping and re-sequencing techniques are required to identify the functional risk-associated variants. Combined with quantitative trait locus analysis, this would aid the identification of target genes and biological pathways involved in keratoconus.

## Supporting information

S1 Table Summary of the regions included in analysis. (XLSX)

S2 Table Coverage of genes reported for the coding regions of the longest transcript included in the captures. Summary of the total coding bases, the total number and percentage of coding bases captured in all capture methods, and the total number of coding bases included in the analysis and the percentage of the captured regions this represents for each gene.
(DOCX)

S3 Table Summary of the potentially pathogenic variants identified and included in analysis. For each variant, the table presents the relevant gene, position, nucleotide and protein changes, the corresponding identification number in dbSNP, the prediction and scores from SIFT and PolyPhen2 (where D = damaging, P = probably/possibly damaging, T = tolerated and B = benign), scaled CADD score, minor allele frequency (MAF) for the ExAC non-Finnish European population and the frequency (freq.) and the number of alternate (alt.) alleles observed in the control and case cohorts in the present study.
(XLSX)
