# Supplementary material for: Rare, potentially pathogenic variants in 21 keratoconus candidate genes are not enriched in cases in a large Australian cohort of European descent
Source: PLoS One. 2018 Jun 20;13(6):e0199178. doi: 10.1371/journal.pone.0199178 (PMC6010250; doi:10.1371/journal.pone.0199178)
Supplement: S2 Table — Summary of the total coding bases, the total number and percentage of coding bases captured in all capture methods, and the total number of coding bases included in the analysis and the percentage of the captured regions this represents for each gene. (DOCX) [file pone.0199178.s002.docx]

| **Gene** | **Transcript** | **Total coding bases in transcript** | **Total coding bases in captured regions (%)** | **Total captured coding bases included in analysis**  **(% of captured regions)** |
| --- | --- | --- | --- | --- |
| *COL4A3* | NM_000091 | 5013 | 4136 (82.5) | 4096 (99.0) |
| *COL4A4* | NM_000092 | 5073 | 4200 (82.8) | 4184 (99.6) |
| *IL1A* | NM_000575 | 816 | 762 (93.4) | 762 (100) |
| *IL1B* | NM_000576 | 810 | 629 (77.7) | 629 (100) |
| *IL1RN* | NM_173841 | 543 | 499 (91.9) | 499 (100) |
| *RAB3GAP1* | NM_012233 | 2946 | 2797 (94.9) | 2797 (100) |
| *TF* | NM_001063 | 2233 | 1683 (75.3) | 1681 (99.9) |
| *FNDC3B* | NM_022763 | 3615 | 3015 (83.4) | 3015 (100) |
| *CAST* | NM_001042442 | 2310 | 1978 (85.6) | 1968 (99.5) |
| *HGF* | NM_000601 | 2187 | 2116 (96.8) | 2116 (100) |
| *IMMP2L* | NM_032549 | 528 | 501 (94.9) | 501 (100) |
| *COL5A1* | NM_000093 | 5517 | 4550 (82.5) | 4548 (100) |
| *NFIB* | NM_005596 | 1263 | 1068 (84.6) | 1068 (100) |
| *MPDZ* | NM_003829 | 6126 | 5534 (90.3) | 5477 (99.0) |
| *RXRA* | NM_001291921 | 1098 | 940 (85.6) | 940 (100) |
| *FOXO1* | NM_002015 | 1968 | 1893 (96.2) | 1561 (82.5) |
| *RAD51* | NM_002875 | 1020 | 928 (91.0) | 928 (100) |
| *BANP* | NM_017869 | 1410 | 818 (58.0) | 573 (70.0) |
| *SLC4A11* | NM_032034 | 2676 | 2610 (97.5) | 2610 (100) |
| *VSX1* | NM_014588 | 1098 | 850 (77.4) | 515 (60.6) |
| *SOD1* | NM_000454 | 465 | 404 (86.9) | 404 (100) |
